# Solving new world animal science problems with a multidisciplinary approach

**DOI:** 10.1093/af/vfab055

**Published:** 2021-10-20

**Authors:** Sue Hatcher, Dianne Mayberry, Stephanie Muir, Michael Campbell, Cara Wilson, Diogo Costa

**Affiliations:** 1 Makin Outcomes Pty Ltd, Orange East, NSW 2800, Australia; 2 School of Agricultural, Environmental and Veterinary Sciences, Charles Sturt University, North Wagga, NSW 2650Australia; 3 CSIRO Agriculture and Food, Queensland Bioscience Precinct, St Lucia, QLD 4067, Australia; 4 Livestock Production, Agriculture Victoria Research, Hamilton, VIC 3300, Australia; 5 School of Health, Medical and Applied Sciences, Central Queensland University, Rockhampton QLD 4701, Australia

Global demand for livestock products is increasing, driven by continued population growth and rising consumer incomes, particularly in emerging economy countries across Africa and Asia. If we are to produce more meat, milk, and eggs, using less resources, and under increased regulatory and consumer pressure, we must understand that livestock systems are intimately interconnected with the environment and play an important role in supporting the livelihoods of billions of people across the supply chain. Livestock production occurs in many forms and environments, ranging from low-input smallholder farms and pastoral systems to technologically advanced and intensive industrial systems. The challenges and opportunities facing these systems are often framed in the context of global megatrends: climate change (Godde et al., 2021), changing consumer preferences and increased public scrutiny on how and where our food is produced (Lawrence et al., 2019), increased global connectedness and trade intensification (Geyik et al., 2021), biosecurity risks (Begley et al., 2019), and the increasing adoption of digital technology (Bahlo et al., 2019). These challenges are significant and complex, and addressing them requires a multidisciplinary approach based on a sound foundation of animal science (Mayberry et al., 2021). This issue of *Animal Frontiers* addresses the key global challenges facing livestock production and highlights opportunities for co-learning and achieving greater impact by working together.

The special issue is contributed by the Australian Association for Animal Sciences (AAAS). As part of our commitment to building capacity in the next generation of animal scientists, this issue has been curated by a team of early and mid-career researchers, with mentoring and support from AAAS fellows.

The first feature paper, by Alders et al. (2021), sets the scene by introducing the multiple and complex dimensions of livestock systems around the world. Understanding the value of and reasons for keeping livestock is essential for designing research, development, and extension programs that will improve income and production efficiency and reduce the environmental impacts of these systems. Livestock are more than just a source of income, food, and fiber for many families; they provide manure and draught power, and have important social and cultural roles, which need to be considered.

Next, Cullen et al. (2021) explore the use of multidisciplinary approaches in creating integrated solutions to climate change adaptation for livestock production. Management of livestock production systems is complex, without the added factors of understanding greenhouse gas mitigation and carbon sequestration. Our authors describe the transdisciplinary approach that they have utilized to explore adaptation options for dairy production systems in Southern Australia. This approach incorporates farmer knowledge, pasture, and animal science with economics, modeling, and social science to develop producer-focused solutions to climate change adaptation. This transdisciplinary approach and the associated research design are highlighted by a case study.

In the third paper, Wynn Mitscherlich et al. (2021) discuss the impact of global trade in animal products on meeting global food security and human nutrition targets. While international trade plays an important role in distributing animal products to high-need communities around the globe, the continuity of supply is often interrupted during times of natural disasters and conflicts when it is most needed. Therefore, domestic supply of animal-sourced foods remains a key element of food security. The key challenge is to support domestic family-based animal production systems while encouraging equitable international trade in animal-sourced foods.

The final feature paper (Jori et al., 2021) provides a holistic overview of the importance and impacts of interactions between wildlife and livestock worldwide, particularly with regard to the biosecurity implications of these interactions. The paper highlights the positive and negative impacts of these interactions for both wildlife and livestock and discusses transmission pathways and strategies to mitigate the impact of interactions between wildlife and livestock. This paper is pertinent considering the current COVID-19 situation, which has awakened the world to the importance of biosecurity and monitoring, measuring, and managing interactions between species.

Three perspectives papers round out the AAAS Special Issue. Rivero et al. (2021) highlight the role of the Global Farm Platform, a multinational and multidisciplinary initiative, in evaluating how diverse livestock production systems around the globe can optimize food quality and quantity while also mitigating the environmental impacts of ruminant production. Masters (2021) and Williams et al. (2021) highlight the opportunities that precision livestock management systems provide to improve both the production and welfare of extensively grazed livestock, while also indicating the challenges in designing systems that are fit for purpose. Masters (2021) advocates for a new paradigm based on a consultative process involving a multidisciplinary approach to increase adoption and realize the transformational opportunities precision livestock management systems have the capacity to provide. The capacity of precision livestock management to enable livestock enterprise management decisions based on whole-of-system production data is outlined by Williams et al. (2021) who highlight opportunities for access to new markets and incentive programs related to climate change and carbon farming.

## About the Authors

**Sue Hatcher** is the Director and Principal Scientist at Makin Outcomes, an independent research and development consultancy specializing in data analysis and reporting, scientific reviews and applied genetics, and sheep management research and consulting. She holds a B.Agric Sci (Hons) and a PhD from The University of Western Australia and is an AAAS Fellow. She has worked in the Australian sheep industry for most of her career and has diverse research interests ranging from genetic improvement of wool production, wool quality and reproductive performance, wool processing, wool metrology and fiber identification, breeding ewe management, and lamb and weaner survival.

**Dianne Mayberry** applies a background in ruminant nutrition to systems-scale analyses that aim to improve the productivity, profitability, and sustainability of livestock production in Australia and emerging economy countries. After completing a BSc and PhD at the University of Western Australia, she spent a year volunteering as an agronomist in the Philippines, before taking up a postdoc at the University of Queensland. She is currently a Senior Research Scientist with the Commonwealth Scientific and Industrial Research Organisation (CSIRO) and leads a multidisciplinary team delivering projects across northern Australia, South and East Asia, and Sub-Saharan Africa.

**Stephanie Muir** is a research scientist at Agriculture Victoria, Department of Jobs, Precincts and Regions, Victoria Australia. Stephanie completed a B. Ag Sci (Hons) and PhD at the University of Melbourne, focused on Greenhouse gas emissions from Australian beef cattle feedlots. She worked as a research assistant in Dairy Production Systems at the University of Melbourne prior to commencing her current role with Agriculture Victoria. Her current research interests cover sheep, beef, and dairy cattle nutrition, with a focus on interaction between forages and animal production, greenhouse gas emissions, feed efficiency, and the use of technology to manage feed intake.

**Michael Campbell** is a lecturer in Farming Systems (with a focus on Meat Science and Ruminant Production) at Charles Sturt University in Wagga Wagga. He completed his PhD in Dairy Farm Systems (USyd) and has an MBA (UNE) and BScAgr (USyd). He has a strong interest in commercial agricultural production and has previously managed a fully integrated cattle operation in Papua New Guinea; he worked in rural banking and as a beef cattle extension officer for the NSW Department of Primary Industries. He has also worked as a consultant on farm business benchmarking projects. He is a member of the Australian Intercollegiate Meat Judging Competition committee and the President of AAAS.

**Cara Wilson** is a Research Officer in the Precision Livestock Management team at Central Queensland University in Rockhampton. Her research focuses on the utilization of AgriTech Technologies in livestock traceability systems and using these technologies to detect livestock health and welfare issues throughout the value chain. Integrating data from pre-farm gate to abattoir and beyond has the potential to improve our biosecurity systems and market access. She also works as a STEM Project Officer where she develops and implements programs centered around getting children excited about STEM and thinking about careers in STEM. She has completed a BEqSci, BSc (HonsI), and her PhD at Charles Sturt University in Wagga Wagga. Her passions are conducting research that provides practical and sustainable outcomes to industry and sharing the results with the wider community.

**Diogo Costa** is a Senior Research Officer at Central Queensland University. His passion for agriculture and livestock led him to study agronomy at the University of Sao Paulo (USP). His involvement with research started there and continued to develop over the years with a Masters at USP and a PhD at the University of Queensland. Over the past 20 yr, he has developed extensive experience in a research career focusing on cattle production in grazing and feedlot systems. He is currently working on a project looking into the use of cutting-edge technology for sensing calving and calf loss. Gathering data to help producers address perinatal calf mortality remains a significant challenge in extensive grazing landscapes and his main goal is to evaluate and enhance tools and systems to provide this critical missing information, working directly with producers to identify their needs, and deliver practical solutions to the livestock industries.
